# Research on identification of key genes and immune–metabolic mechanisms in atrial fibrillation through integrated multi-cohort transcriptomic analysis and machine learning

**DOI:** 10.1097/MD.0000000000049463

**Published:** 2026-06-26

**Authors:** Mierzhati Maimaiti, Aizizha Paerhati, Shenhong Liu, Xianglin Du, Wen Bai

**Affiliations:** a Urumqi Friendship Hospital, Tianshan District, Urumqi, Xinjiang, China; d Cardiovascular Center, Urumqi Friendship Hospital, Tianshan District, Urumqi, Xinjiang, China; b Department of Respiratory and Critical Care Medicine, Urumqi Friendship Hospital, Tianshan District, Urumqi, Xinjiang, China; c Department of Critical Care Medicine, Urumqi Friendship Hospital, Tianshan District, Urumqi, Xinjiang, China

**Keywords:** atrial fibrillation, functional enrichment analysis, gene expression, immune cell infiltration, machine learning

## Abstract

This study aimed to integrate multiple datasets for the identification of atrial fibrillation (AF)-related differentially expressed genes (DEGs), analyze their underlying mechanisms through functional enrichment and machine learning, construct diagnostic models, and explore immune–metabolic interactions to provide novel biomarkers and theoretical foundations. Gene expression datasets were integrated and normalized, with batch effects removed using principal component analysis. Differential expression analysis, functional enrichment analysis (Gene Ontology and Kyoto Encyclopedia of Genes and Genomes pathways), and machine learning-based feature gene selection and model construction were performed. Shapley additive explanations analysis was utilized to interpret the constructed models, while gene set enrichment analysis, gene set variation analysis, and immune cell infiltration analysis were conducted to investigate the associations between feature genes and immune infiltration. After integrating and normalizing gene expression data and eliminating batch effects via principal component analysis, 6 DEGs were identified, including 4 upregulated and 2 down-regulated ones. Functional enrichment analysis showed these DEGs were significantly enriched in neuro-related biological processes and pathways, indicating their key roles in AF pathogenesis. Five key feature genes were selected using LASSO, random forest, and support vector machine-recursive feature elimination algorithms. They had significant expression differences between the AF and control groups (*P* < .001) and were located on distinct chromosomes. The constructed random forest and support vector machine models performed excellently (area under the curve ≥ 0.85). Shapley additive explanations analysis revealed TNNI1 contributed most to model prediction, with its expression significantly positively correlated with immune cell infiltration. Gene set enrichment analysis and gene set variation analysis analyses further showed feature genes participated in AF pathogenesis by regulating immune modulation, metabolic pathways, and autophagy. Immune cell infiltration analysis found altered proportions of T-cell subsets and M0 macrophages in the AF group, along with complex links between feature gene expression and immune cell function. This study systematically elucidated the unique gene expression patterns and key regulatory pathways associated with AF, clarifying the crucial roles of feature genes in immune regulation, metabolic imbalance, and cellular dysfunction. These findings provide a theoretical basis and potential therapeutic targets for understanding AF pathogenesis and developing targeted treatment strategies.

## 1. Introduction

Atrial fibrillation (AF), one of the most prevalent clinical arrhythmias, encompasses multiple pathological processes, including electrical remodeling, structural remodeling, and neural remodeling, which significantly impair the quality of life and prognosis of approximately 33.5 million patients globally.^[1,2]^ While prior studies have demonstrated that abnormal ion channels in myocardial cells, fibrosis, and inflammatory responses play critical roles in AF development, the dysregulation of gene expression networks and their mediation of immune–metabolic interactions warrant further investigation.^[3,4]^ The advent of high-throughput sequencing technologies has enabled the analysis of differentially expressed genes (DEGs), offering novel insights into the molecular mechanisms underlying AF. Research has revealed substantial alterations in gene expression profiles within the atrial tissues of AF patients, such as mutations in SCN5A, a gene associated with ion transport, which can disrupt sodium channel function and thereby induce arrhythmia.^[5]^ Nevertheless, the functional characterization of individual genes is insufficient to elucidate the intricate pathological network of AF. Therefore, it is imperative to identify key characteristic genes through integrative analyses of multiple datasets and to establish a “gene–pathway–phenotype” regulatory framework.

In recent years, the integration of machine learning algorithms into the biomedical field has markedly improved the efficiency and accuracy of disease biomarker identification. For example, the random forest (RF) algorithm has been effectively utilized for AF risk prediction, achieving an area under the curve (AUC) value of 0.82 based on blood biomarkers.^[6]^ Additionally, immune cell infiltration analysis has elucidated the dynamic alterations in the immune microenvironment during AF. Specifically, M1 polarization of macrophages promotes atrial fibrosis via tumor necrosis factor-α secretion, while the imbalance of T-cell subsets is closely associated with electrical remodeling.^[7,8]^ These observations indicate that aberrant gene expression may contribute to AF progression by modulating immune–metabolic pathways; however, the precise molecular mechanisms underlying this process require further systematic investigation.

It is noteworthy that several bioinformatics studies on AF have been published in recent years. Common analytical strategies include differential expression analysis coupled with protein–protein interaction network construction or weighted gene co-expression network analysis applied to single or limited Gene Expression Omnibus (GEO) cohorts. Others integrate weighted gene co-expression network analysis with machine learning-based feature selection methods – such as LASSO regression or support vector machine-recursive feature elimination (SVM-RFE) – to identify candidate biomarkers. A subset of studies further incorporates proteomic, genomic, or other multi-omics data to delineate tissue-specific molecular landscapes in AF.^[9–11]^ Nevertheless, key methodological limitations persist across prior work, particularly in: standardized preprocessing and harmonization of cross-platform cohort data; robustness of feature selection – specifically, mitigation of algorithmic bias arising from reliance on a single method; and integration of model interpretability with biologically grounded mechanistic validation – especially regarding immune–metabolic crosstalk.^[12,13]^

Accordingly, this study advances beyond existing approaches in 3 interrelated dimensions:

First, at the data level, we systematically integrated multiple independent GEO cohorts – spanning diverse platforms and sample sources – and applied uniform probe-to-gene annotation, batch effect correction (via ComBat), and quantile normalization. This harmonized pipeline enhances cross-study comparability and strengthens the reproducibility and generalizability of findings. Crucially, by prioritizing DEGs consistently identified across ≥3 independent cohorts, we substantially reduce platform- or cohort-specific artifacts and increase confidence in biological relevance.^[14]^

Second, at the methodological level, we implemented a consensus-driven feature selection framework: LASSO, SVM-RFE, and RF were independently applied, and only genes retained in the intersection were advanced for downstream modeling. This strategy synergistically leverages linear sparsity, margin-based classification, and ensemble nonlinear learning, thereby improving stability, reducing overfitting, and enhancing biological plausibility. Subsequently, multiple supervised classifiers were benchmarked for diagnostic performance, and shapley additive explanations (SHAP) values were computed to deliver transparent, sample-level interpretations – ensuring that predictive features are both discriminative and biologically interpretable.

Third, at the mechanistic level, we bridged computational prediction with functional insight by jointly analyzing: pathway enrichment dynamics (via gene set enrichment analysis [GSEA] and gene set variation analysis [GSVA]); immune cell composition inferred from bulk RNA-seq using CIBERSORTx; and correlations between hub genes and immune–metabolic pathway activities. This integrative, multi-tiered analysis – from gene to pathway to cellular context – yields a testable, hypothesis-generating framework for immune–metabolic interplay in AF pathogenesis.^[15]^

Collectively, this study aims to: identify robust, cross-cohort-validated DEGs associated with AF and characterize their functional roles; develop and rigorously validate a clinically translatable diagnostic model grounded in these molecular features; and elucidate how dysregulated gene expression orchestrates immune–metabolic reprogramming in AF. Our findings provide a mechanistically informed foundation for precision diagnostics and targeted therapeutic development. Importantly, current AF clinical guidelines underscore the urgent need for improved risk stratification and early detection tools, reinforcing the translational significance of identifying and validating molecular signatures for routine clinical implementation.^[16]^

## 2. Materials and methods

### 2.1. Data sources and preprocessing

Gene expression datasets associated with AF were retrieved from the GEO database (https://www.ncbi.nlm.nih.gov/geo/). Specifically, the following datasets were included: GSE31821 (4 AF patients and 2 controls), GSE79768 (13 AF patients and 13 controls), GSE108660 (5 AF patients and 5 controls), GSE115574 (15 AF patients and 15 controls), and GSE143924 (15 AF patients and 15 controls). All datasets were profiled using either the Agilent or Affymetrix platform, and all samples were derived from human subjects. This study involves no ethical concerns and adheres to relevant research guidelines.

To enhance reproducibility and ensure transparent, criteria-driven dataset inclusion, we systematically curated key metadata, including microarray platform (Affymetrix or Agilent), biological source (tissue or cell type), and clinical group assignment for each GEO dataset (see Table S1, Supplemental Digital Content 1). All included datasets comprise human transcriptome expression profiles generated using Affymetrix or Agilent microarray platforms, and samples are clearly annotated as either AF or non-AF controls.

With respect to tissue specificity, this study exclusively incorporates transcriptomic data derived from anatomically relevant cardiac tissues – specifically, left/right atrial myocardium and adjacent structures such as epicardial adipose tissue – to capture localized pathophysiological alterations and the immune–metabolic microenvironment intrinsic to AF and postoperative AF. In contrast, datasets originating from systemic compartments (including peripheral blood, plasma, serum, PBMCs) or immortalized cell lines were excluded to reduce tissue-of-origin confounding and to focus on cardiac-specific mechanisms.

Inclusion criteria: human-derived (*Homo sapiens*) transcriptomic datasets; samples obtained from anatomically relevant cardiac tissues (including atrial myocardium and adjacent structures) with clear assignment to either AF or non-AF control groups as defined in the original GEO metadata/publications; availability of raw data or preprocessed expression matrices together with platform-specific annotation files enabling reliable probe-to-gene symbol mapping; minimum sample size of n ≥ 2 per group; and no evidence of duplicate samples.

Exclusion criteria: datasets derived from non-cardiac tissues (e.g., peripheral blood, serum, plasma, PBMCs), immortalized cell lines, or non-human models; absence of definitive clinical grouping metadata or incomplete/inconsistent sample-level annotations; unavailability of platform annotation files or failure to achieve high-confidence probe-to-gene symbol mapping; and cohorts comprising heterogeneous or poorly defined subtypes. Notably, publicly available surgical cohorts (including those with underlying valvular heart disease or coronary artery bypass grafting) were retained when atrial tissue was explicitly sourced and annotated. Potential confounding related to surgical context was mitigated through batch correction during integration, and its potential impact on biological interpretation was explicitly addressed in the Discussion (with sensitivity analyses considered where appropriate). Samples exhibiting severe technical artifacts that persisted after standardized quality control and could not be adequately corrected using established normalization or filtering procedures were also excluded.

### 2.2. Data integration and standardization

The raw data were imported and preprocessed using the GEOquery and limma packages in R software (v4.2.3). Specifically, the following steps were performed: probe IDs were converted to gene symbols according to the platform annotation file; when multiple probes mapped to the same gene, the average expression value was calculated. Batch effects were removed using the ComBat algorithm from the sva package. Quantile normalization was conducted using the normalizeQuantiles function in the limma package. Finally, low-expression genes (with expression levels < 100 in ≥ 80% of the samples) were excluded from further analysis.

### 2.3. Differential expression analysis

Differential expression analysis was performed on the integrated dataset. A linear model was constructed using the limma package to identify DEGs between the AF group and the control group. The screening criteria were set as follows: adjusted *P*-value (false discovery rate [FDR]) < 0.05 and |log2FC| > 1. The EnhancedVolcano package was utilized to generate a volcano plot for visualizing the DEGs. Additionally, heatmap clustering analysis was conducted to illustrate the differences in expression patterns among samples.

### 2.4. Functional enrichment analysis

The Metascape online tool (https://metascape.org) was employed to perform systematic functional annotation and pathway enrichment analysis of the DEGs. The biological significance of the DEGs was elucidated through 3 aspects of Gene Ontology (GO) enrichment analysis: biological process, cellular component, and molecular function. Kyoto Encyclopedia of Genes and Genomes (KEGG) pathway analysis was utilized to identify pathways associated with AF pathology, while Reactome was applied to complement the analysis of biological processes not covered by KEGG. Reliable results were filtered based on 3 criteria: *P* < .01, a minimum of 3 DEGs in a single functional category, and an enrichment factor >1.5. Cytoscape (v3.9.1) was used to construct an enrichment network, where functional categories served as nodes and gene-sharing relationships served as edges. The hierarchical relationships of functional clustering were visualized using node size, color intensity, and edge weight, thereby presenting the functional regulatory network of DEGs in AF.

### 2.5. Feature gene selection via machine learning

Three machine learning algorithms were utilized to further identify the key feature genes: LASSO regression: the glmnet package was employed, and the optimal λ value was determined using 10-fold cross-validation; the SVM-RFE algorithm, for which the e1071 package was used to perform recursive feature elimination, with the feature of the lowest weight removed in each iteration; and RF, for which the randomForest package was applied to compute feature importance, with ntree set to 1000. The intersection of the screening results from these 3 methods was selected as the final feature gene set. Additionally, the VennDiagram package was used to generate a Venn diagram to visualize the intersection relationship.

### 2.6. Visualization of feature genes

The screened feature genes were subjected to multidimensional visualization analysis to intuitively present their biological characteristics and expression patterns. Bar plots were utilized to compare the expression levels of feature genes between the AF group and the control group, with *t* tests conducted to indicate significant differences (*P* < .05), thereby visually demonstrating the upregulation or down-regulation trends of the genes. Based on the UCSC Genome Database, the circlize package in R was employed to generate chromosome circle plots (Circos Plots). The outer circle annotated chromosome positions, while the inner circle displayed gene expression intensities in heatmap format, providing a clear visualization of the chromosomal distribution patterns of feature genes and differences in expression abundance. Additionally, the pheatmap package was used to construct heatmaps, where hierarchical clustering based on Euclidean distance was performed for both samples and genes, revealing the expression patterns of feature genes across different samples. The depth of color reflected the level of expression, and the clustering dendrogram highlighted the similarities and differences in expression among samples.

### 2.7. Construction and validation of machine learning models

AF diagnostic models were constructed using multiple machine learning algorithms, and their classification performance was systematically evaluated. The specific methods are as follows: based on the feature gene set, 5 supervised learning algorithms were employed for modeling: RF was implemented using the randomForest package, with 1000 decision trees and mtry set to the square root of the number of features for sampling; out-of-bag data was utilized to estimate the error. SVM was modeled using the RBF kernel in the e1071 package, with grid search employed to optimize the parameters C (ranging from 10^−3^ to 10^3^) and γ (ranging from 10^−3^ to 10^1^). Logistic Regression was constructed using the glm package, with L1 regularization introduced and optimized via glmnet. *K*-nearest neighbor used the class package to search for the optimal number of neighbors (*k* ranging from 1 to 20), with Euclidean distance serving as the similarity metric. Neural network (NeuralNet) was modeled using the neuralnet package to construct a multi-layer perceptron with a single hidden layer (the number of nodes being half the number of features); the activation function was set to tanh, and the model underwent 1000 iterations.

### 2.8. SHAP interpretability analysis

The SHAP method was employed to elucidate the decision-making mechanism of the model. Using the shapr package in R, based on the trained machine learning model (e.g., RF), 1000 Monte Carlo simulations were conducted to calculate the SHAP values for each feature gene’s contribution to the prediction outcome, thereby quantifying its marginal contribution to the model output. A feature importance bar chart was generated, sorted by the mean of the absolute SHAP values, to display the contribution weights of each feature gene to the model prediction, enabling the intuitive identification of key driver genes. A scatter plot was constructed with the feature gene expression level on the *x*-axis and the SHAP value on the *y*-axis, with a local regression curve fitted to reveal the nonlinear relationship between gene expression and model prediction (e.g., threshold effects or biphasic regulation). Representative samples were selected, and the prediction logic for individual samples was visualized using force plots: each feature gene’s SHAP value was represented as an arrow, with positive contributions (promoting AF prediction) indicated in red and negative contributions (inhibiting AF prediction) in blue. The length of the arrow reflected the intensity of the influence, providing an intuitive demonstration of the combined effect of multi-gene interactions on the prediction outcome.

### 2.9. GSEA and GSVA analysis

GSEA: Enrichment analysis was performed on predefined gene sets (MSigDB v7.5.1) using the clusterProfiler package to identify the overall enrichment trends of DEGs in biological functions. DEGs were ranked in descending order based on log2FC and used as the input gene list for analysis. Enrichment analysis was conducted on 3 major categories of gene sets: Hallmark (signature pathways), KEGG (Kyoto Encyclopedia of Genes and Genomes), and GO (Gene Ontology). Enrichment scores (NES) were calculated through 1000 permutations, and a significant enrichment threshold of FDR < 0.05 was applied to screen core functional pathways.

GSVA: A non-parametric method was utilized to evaluate sample-specific pathway activity changes. The GSVA package was employed to calculate enrichment scores for each sample across predefined gene sets, generating a pathway activity matrix. A linear model was constructed using the limma package to compare pathway activity differences between the AF group and the control group (adjusted *P* < .05). Additionally, Spearman correlation analysis was performed to investigate the associations between significantly altered pathways and clinical phenotypes (e.g., disease duration and severity), thereby uncovering potential functional regulatory networks.

### 2.10. Immune cell infiltration analysis

The CIBERSORT algorithm (https://cibersort.stanford.edu) was employed to estimate the relative proportions of 22 immune cell types in the samples. The normalized gene expression matrix was input into the algorithm, and 1000 permutation tests were performed to obtain reliable estimation results with a significance threshold of *P* < .05. The pheatmap package was utilized to generate an immune cell infiltration heatmap, while the ggplot2 package was used to create violin plots and box plots for comparing the differences in immune cell composition between the AF group and the control group. Additionally, the Spearman correlation coefficients among immune cells were calculated, and a correlation network was constructed to visualize these relationships.

### 2.11. Correlation analysis of feature genes and immune infiltration

The Spearman correlation between the expression levels of feature genes and the infiltration levels of immune cells was calculated. The ggpubr package was utilized to generate scatter plots and fit linear regression models. The association heatmap of feature genes and immune cells was presented using the pheatmap package. An immune-related risk score model was constructed, and its diagnostic value for AF was evaluated.

## 3. Results

### 3.1. PCA analysis

**Figure 1.**
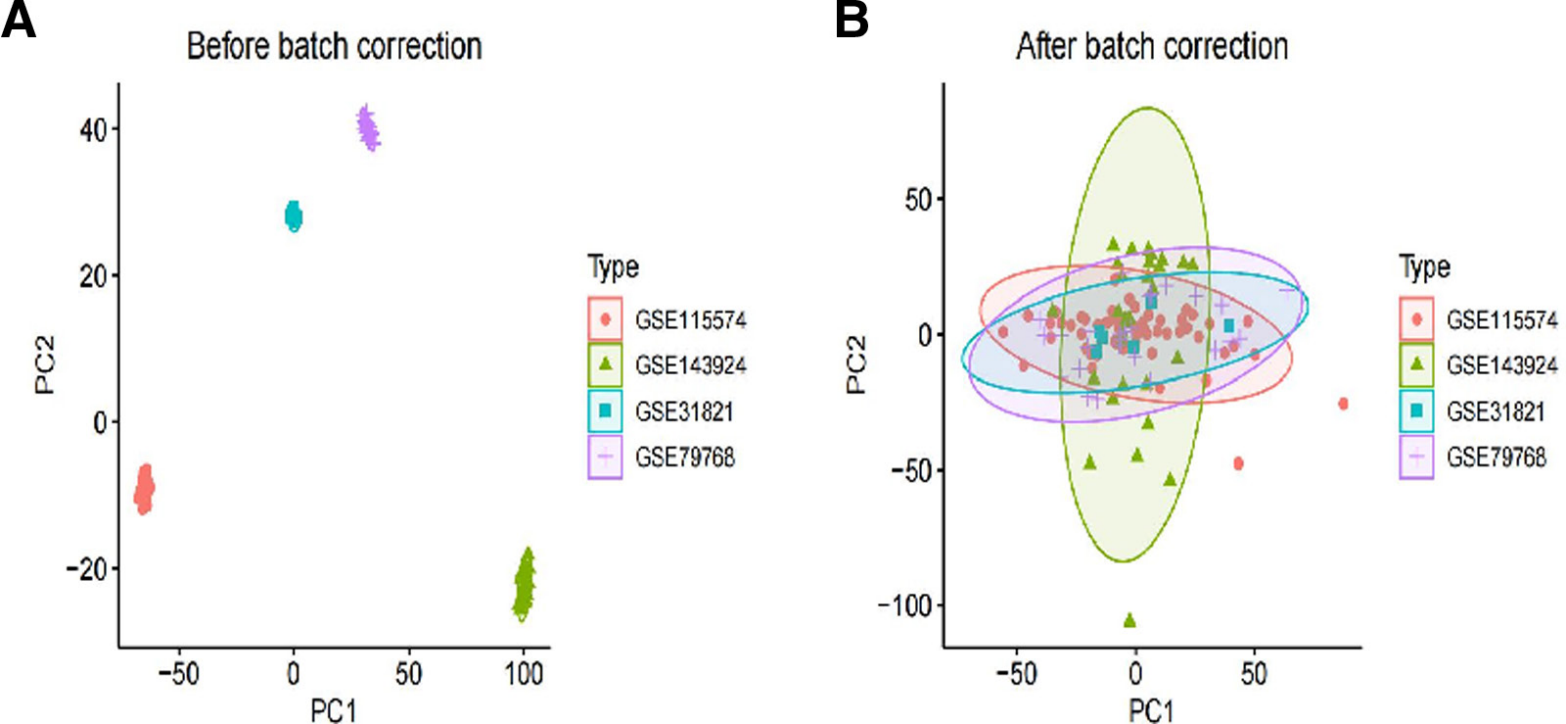
PCA analysis before and after batch correction. (A) PCA plot before batch correction; (B) PCA plot after batch correction. PCA = principal component analysis.

Principal component analysis (PCA) was conducted on the integrated and standardized gene expression dataset to assess the overall variability among samples and the effectiveness of batch correction. The results demonstrated that prior to batch correction, the range of the PC1 axis was −50 to 100, while that of the PC2 axis was −20 to 40. Sample variation was relatively low, and the AF group and control group exhibited no clear clustering. Additionally, some datasets showed scattered sample distributions, with samples from datasets such as GSE115574 displaying block-like patterns. This indicated that the original data were significantly influenced by batch effects. Following batch correction, the PCA revealed that the range of the PC1 axis was approximately −50 to 50, and that of the PC2 axis was approximately −100 to 50. PC1 captured more pronounced differences between groups. In the corrected PCA plot, the AF group and control group were clearly separated, with samples from datasets such as GSE79768 forming distinct clusters within their respective groups and exhibiting clear boundaries between groups. Samples from different datasets were uniformly distributed within groups, confirming the effectiveness of the ComBat algorithm in eliminating batch effects (Fig. 1A and B).

### 3.2. Differential expression gene analysis

**Figure 2.**
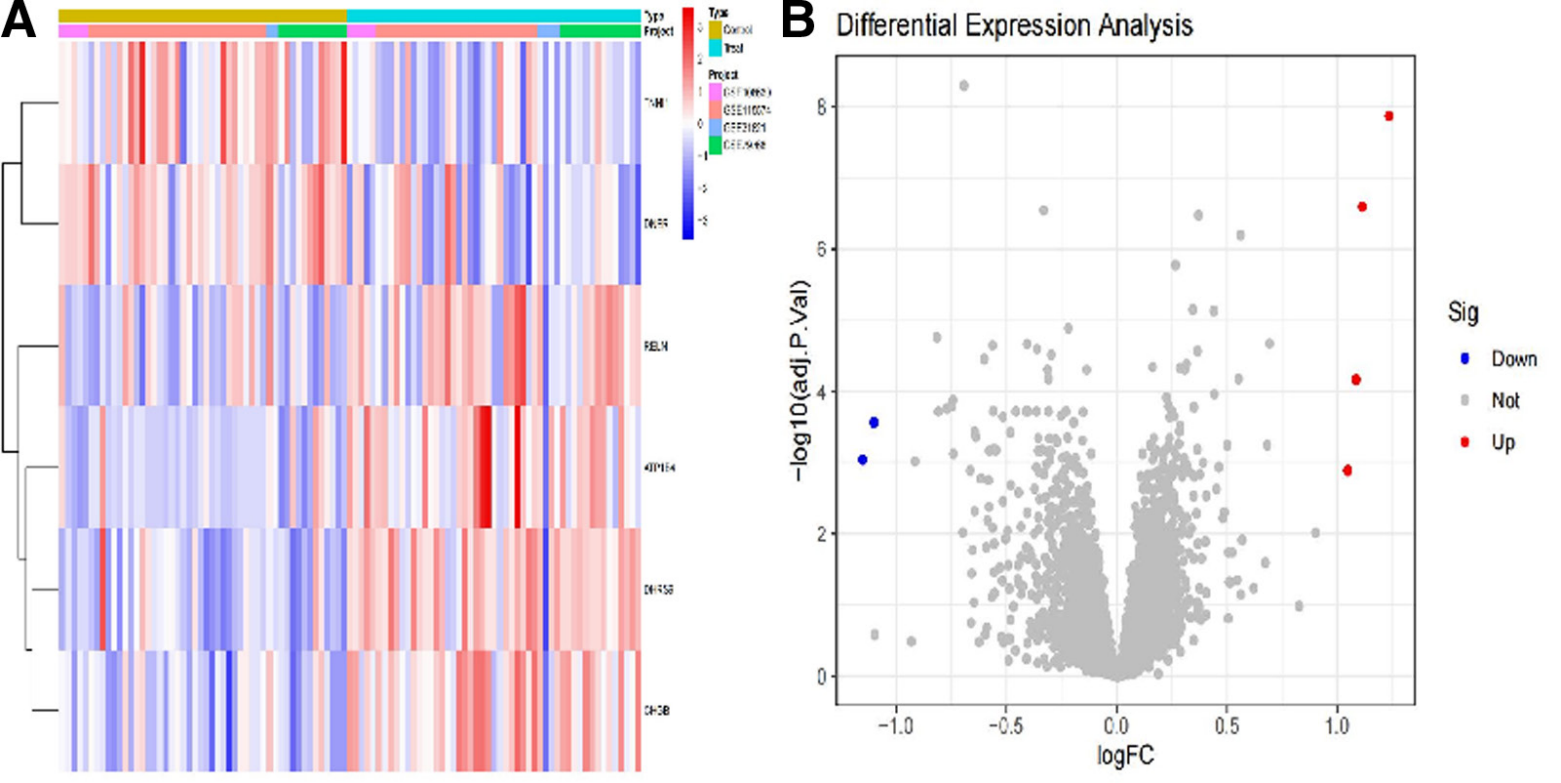
Differential expression analysis. (A) Heatmap of differentially expressed genes; (B) Volcano plot of differentially expressed genes.

Following the confirmation of data quality through PCA analysis, differential expression analysis was performed on the integrated dataset. A total of 6 DEGs were identified between the AF group and the control group, comprising 4 upregulated genes and 2 downregulated genes (Table 1). The volcano plot visually illustrated the distribution of DEGs, whereas the heatmap clustering analysis distinctly revealed the significant differences in gene expression patterns between AF samples and control samples. The clustering dendrogram demonstrated that the samples from the 2 groups clustered separately, further corroborating the unique gene expression profile in the AF group when combined with the PCA analysis results (Fig. 2A and B).

**Table 1:** Results of DEGs.

Id	logFC	AveExpr	t	*P*-value	adj.P.Val	*B*
DHRS9	1.23494266666666	8.66776791089109	7.99965488641177	1.97168755638413e-12	1.35799980445957e-08	17.6275594027004
CHGB	1.11275309294118	8.25180568514851	7.32500365139828	5.57154752240582e-11	2.558268904038e-07	14.5130617648146
ATP1B4	1.08563905921569	4.25036166039604	5.66566745545338	1.34529947696605e-07	6.86351862785457e-05	7.25244208767709
TNNI1	−1.100940295	6.66496631287129	−5.206274443	9.93615477089173e-07	0.000273741063938067	5.39521651931003
DNER	−1.152065165	6.41799826435644	−4.759827875	6.36503987778581e-06	0.000913316919963537	3.6763663176408
RELN	1.04723875607843	7.37641506831683	4.62884194134245	1.07831457503554e-05	0.00129163332792301	3.18997801694991

DEGs = differentially expressed genes, LogFC = log fold change.

### 3.3. Functional enrichment analysis

**Figure 3.**
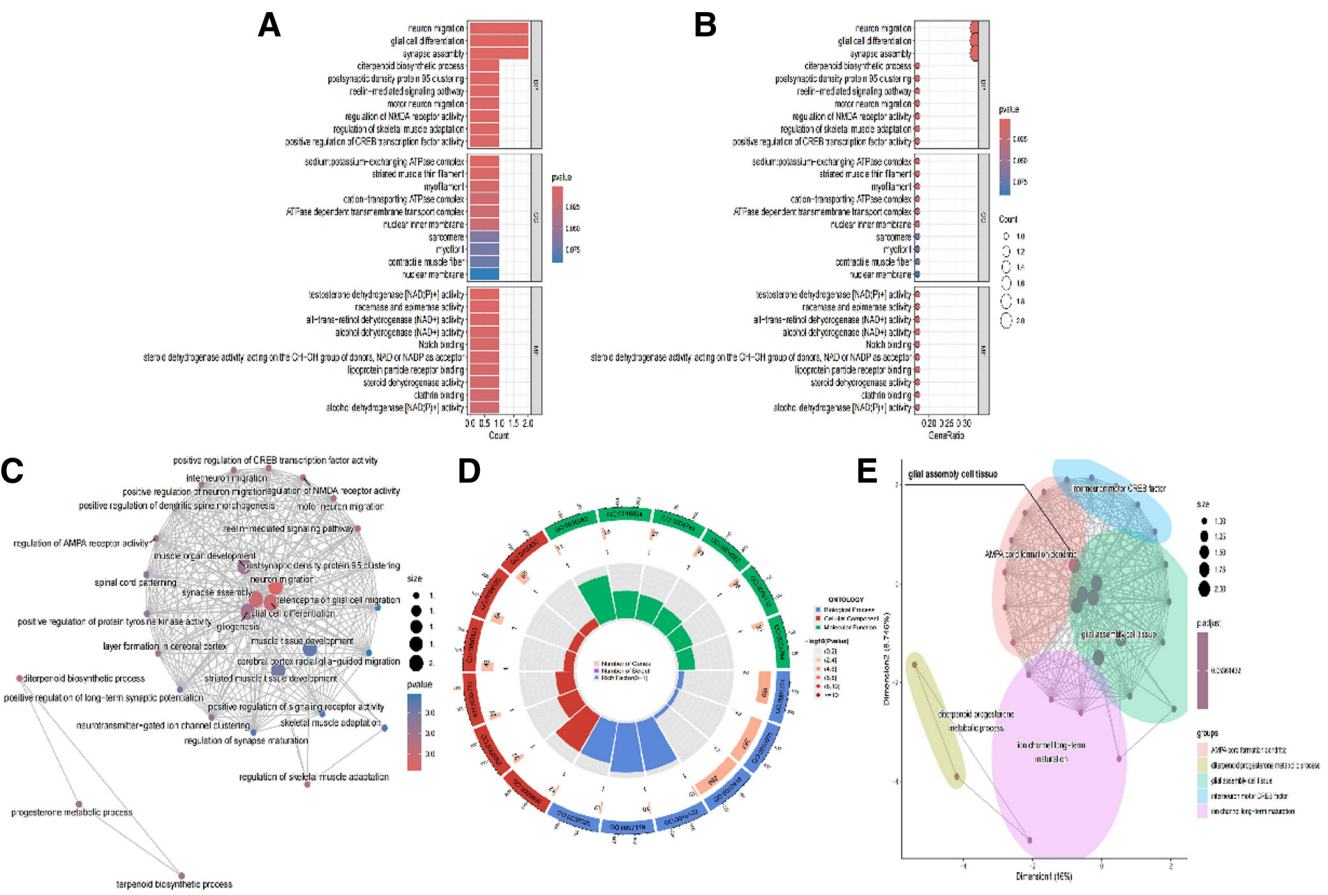
GO functional enrichment analysis. (A) GO enrichment bar plot; (B) GO enrichment dot plot; (C) GO enrichment network plot; (D) GO enrichment chord plot; (E) GO functional clustering bubble plot. GO = Gene Ontology.

Functional enrichment analysis was conducted as an in-depth investigation of the DEGs identified, aiming to explore their primary enrichment patterns in biological processes (BP), cellular components, and molecular functions (MF). This analysis further elucidates their potential roles in underlying biological mechanisms. Bar enrichment plots (first 2 plots): in the BP category, functional terms such as “neuron migration” (neuronal migration) and “glial cell differentiation” (differentiation of glial cells) were significantly enriched, suggesting that these biological processes may play critical roles in gene expression changes associated with AF. In the cellular components category, terms related to cellular components, such as “sodium:potassium – exchanging ATPase complex” (sodium–potassium exchanging ATPase complex), were observed to be enriched. In the molecular function category, MF like “testosterone dehydrogenase [NAD(P)+] activity” (testosterone dehydrogenase activity) were significantly enriched. Enrichment network bubble plot: the plot revealed that nodes corresponding to biological processes such as “neuron migration,” “glial cell differentiation,” and “synapse assembly” (assembly of synapses) were centrally located within the network and exhibited a deeper red coloration, indicating a higher number of associated genes. These findings suggest that these processes represent core enriched functions in the gene function network of AF and may exhibit close interconnections, collectively participating in relevant biological mechanisms. GO enrichment chord plot: this visualization allows for a direct comparison of the enrichment levels of GO terms across different categories. In the BP category, certain terms demonstrated higher enrichment degrees, reflecting their importance in the gene functions associated with AF. Additionally, this plot clearly highlights differences in the number of enriched genes among various functional categories. Functional clustering bubble plot: the plot illustrates the distribution of functional terms within distinct functional clustering groups. For instance, in the “glial assembly cell tissue” (assembly of glial cell tissue) clustering group, multiple functional terms were identified, with some nodes appearing darker in color, indicating significant enrichment within this cluster. Furthermore, potential functional associations or differences among different clustering groups were observed, suggesting their collective involvement in AF-related biological processes. Overall, the results indicate that neuro-related biological processes (e.g., neuronal migration, glial cell differentiation) and certain muscle-related cellular components and MF may play pivotal roles in the onset and progression of AF (Fig. 3A–E).

**Figure 4.**
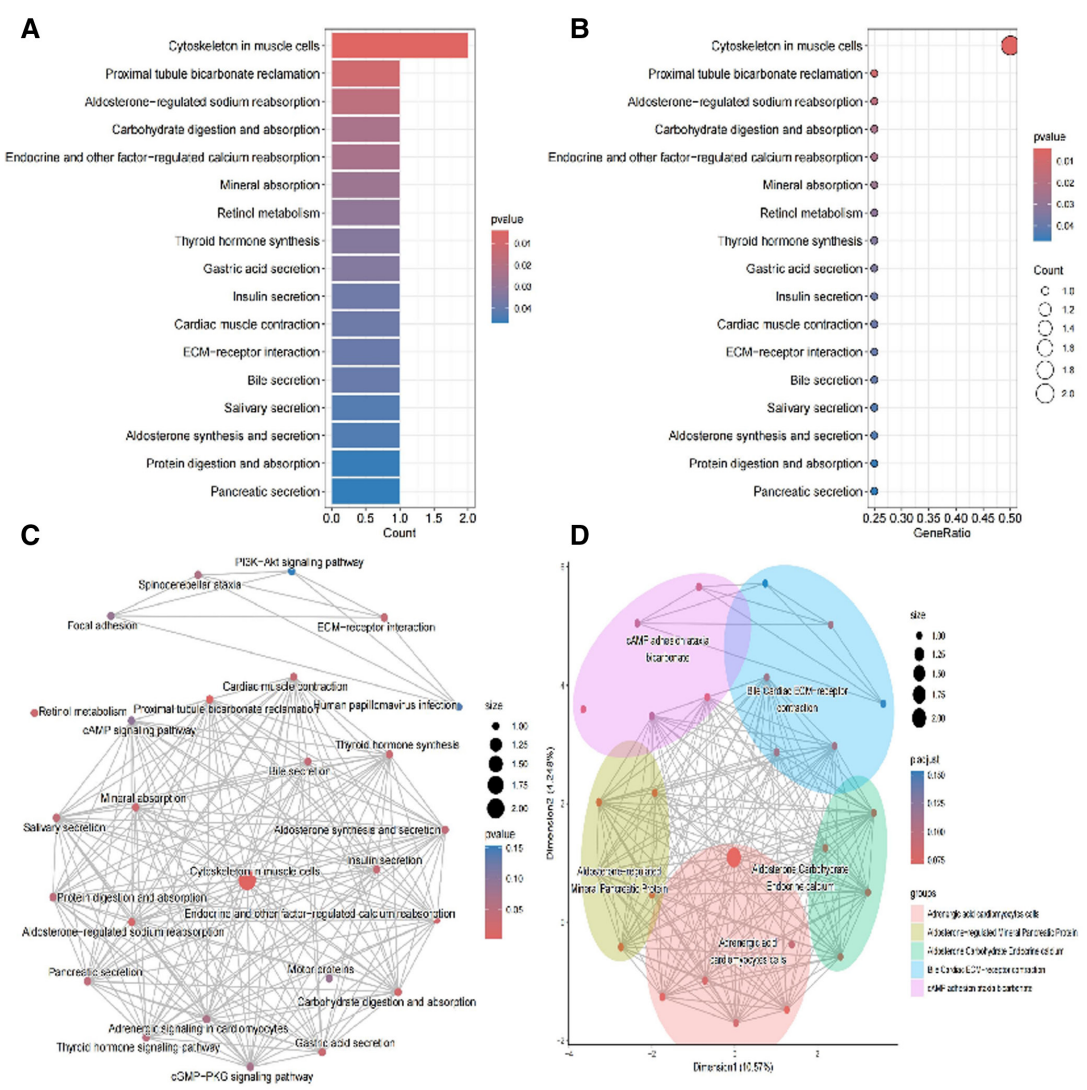
KEGG pathway enrichment analysis. (A) KEGG enrichment bar plot; (B) KEGG enrichment dot plot; (C) KEGG enrichment network plot; (D) KEGG functional clustering bubble plot. KEGG = Kyoto Encyclopedia of Genes and Genomes.

KEGG pathway enrichment analysis serves as a critical approach to investigate the biological pathways in which DEGs are involved, thereby aiding in the elucidation of potential molecular mechanisms underlying diseases. Bar enrichment plots (first 2 plots): the “cytoskeleton in muscle cells” pathway exhibited the highest degree of enrichment with the smallest *P*-value (indicated by the darkest color) and a substantial number of enriched genes, suggesting its pivotal role in the functional aspects of AF-related genes. Additionally, pathways such as “proximal tubule bicarbonate reclamation” and “aldosterone-regulated sodium reabsorption” demonstrated significant enrichment, indicating their potential associations with AF. Enrichment network bubble plot: in this plot, the “cytoskeleton in muscle cells” node was notably large and red, reflecting a high number of enriched genes and significant enrichment, positioning it at the core of the network. Pathways such as “PI3K–Akt signaling pathway” and “ECM–receptor interaction” were observed to have extensive connections with other pathways, implying their roles as signal transduction hubs or functional association bridges within the KEGG pathway network related to AF, collectively participating in the pathophysiological processes of AF. Functional clustering bubble plot: each clustering group encompassed multiple KEGG pathways. For instance, in the “adrenergic acid cardiomyocytes cells” group, certain nodes appeared darker in color and were significantly enriched, highlighting the importance of these pathways in the functional aspects of AF-related genes. Intersections and connections between different clustering groups were evident, suggesting that these pathways do not function independently but rather collaborate or influence 1 another, jointly contributing to the biological mechanisms of AF. Overall, through this series of KEGG pathway enrichment analysis plots, it is evident that DEGs between the AF group and the control group are significantly enriched in multiple biological pathways. Pathways associated with the cytoskeleton of muscle cells, ion transport, and endocrine regulation may play crucial roles in the onset and progression of AF. These pathways are interrelated, forming a complex network that collectively influences the pathological process of AF (Fig. 4A–D).

### 3.4. Machine learning-based feature gene selection and visualization

#### 3.4.1. Feature gene selection

**Figure 5.**
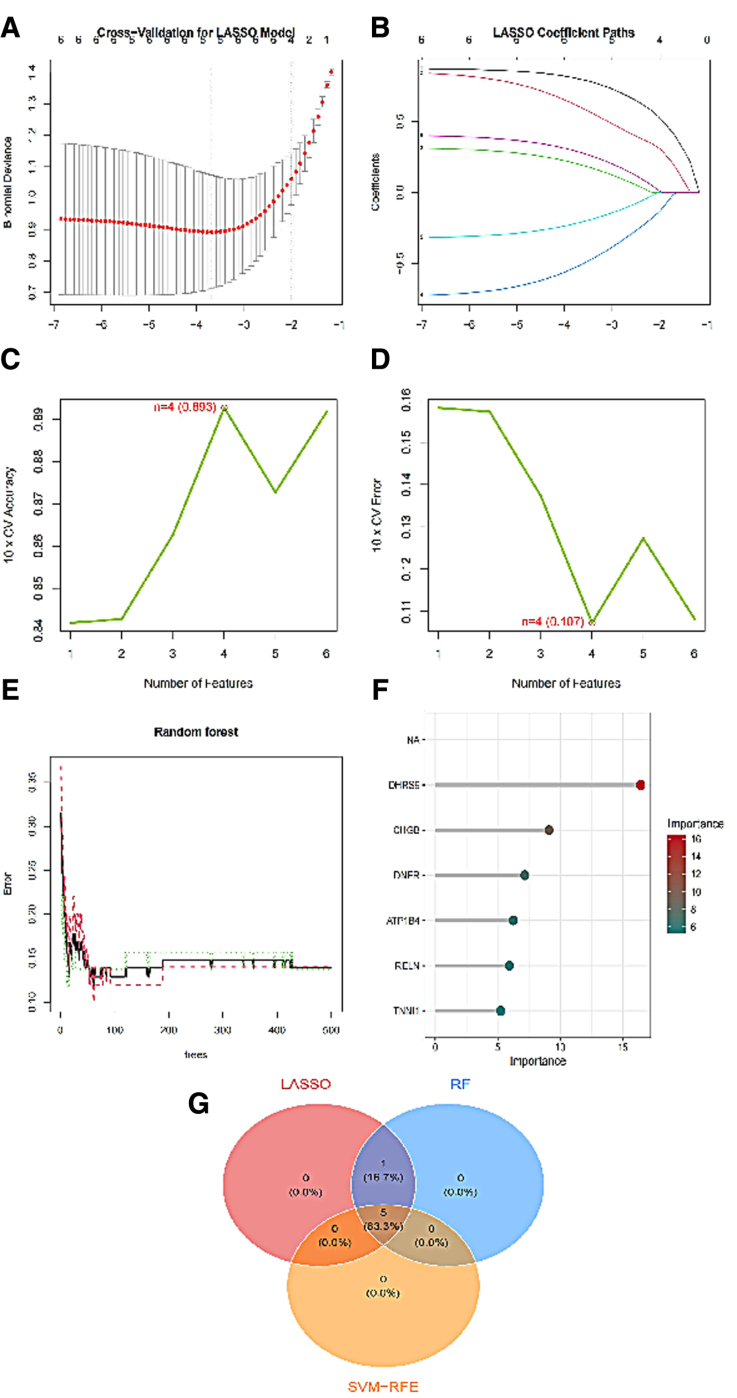
Machine learning-based feature gene selection. (A) LASSO cross-validation curve; (B) LASSO coefficient profile; (C) SVM-RFE accuracy curve; (D) SVM-RFE error curve; (E) random forest error curve; (F) random forest feature importance plot; (G) Venn diagram of feature genes selected by 3 algorithms. SVM-RFE = support vector machine-recursive feature elimination.

The figure presents 3 sets corresponding to LASSO, RF, and SVM-RFE, illustrating the genes selected by each algorithm. The intersection of these sets reveals that all 3 algorithms collectively identified 5 common genes, representing 83.3% of the total number of intersecting genes. Additionally, LASSO and RF jointly identified 1 gene unique to their overlap, accounting for 16.7%. This analysis elucidates the degree of overlap among the selection results from different algorithms and confirms the source of key feature genes (Fig. 5A–G).

#### 3.4.2. Visualization of key feature genes

**Figure 6.**
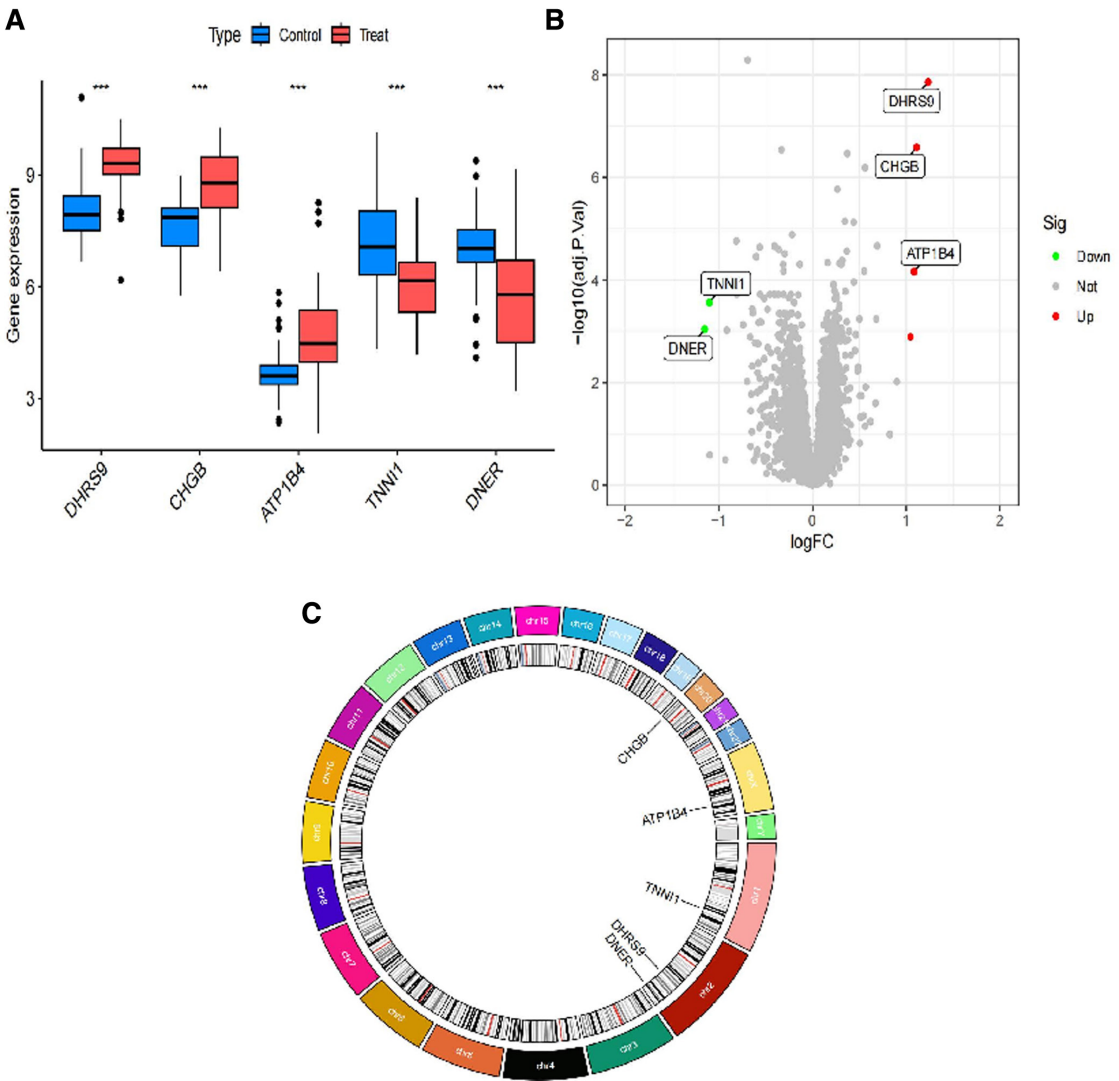
Visualization of key feature genes. (A) Box plots of key feature genes; (B) volcano plot of key feature genes; (C) chromosomal distribution of key feature genes.

Key feature genes were visualized using box plots, volcano plots, and chromosome circle plots, providing insights into the expression differences, statistical significance, and chromosomal localization of these genes between the AF group and the control group from multiple perspectives. The box plots illustrated the expression levels of genes such as DHRS9 and CHGB in both the control and experimental groups. Notably, DHRS9 and CHGB exhibited higher median values in the experimental group, whereas TNNI1 and DNER showed lower median values, with highly significant differences (*P* < .001). The volcano plots, with log fold change and −log10 (adj.P.Val) as coordinates, clearly distinguished upregulated genes (e.g., DHRS9) from downregulated genes (e.g., TNNI1), which was consistent with the trends observed in the box plots. The chromosome circle plots further demonstrated the distribution of these genes across different chromosomes (chr1–chr22). Collectively, these 3 visualization methods provided multidimensional information on expression differences, statistical significance, and chromosomal localization, mutually corroborating and clarifying the upregulation or down-regulation of specific genes. These findings suggest that these genes may play critical roles in the pathogenesis of AF (Fig. 6A–C).

### 3.5. Construction and evaluation of machine learning models

**Figure 7.**
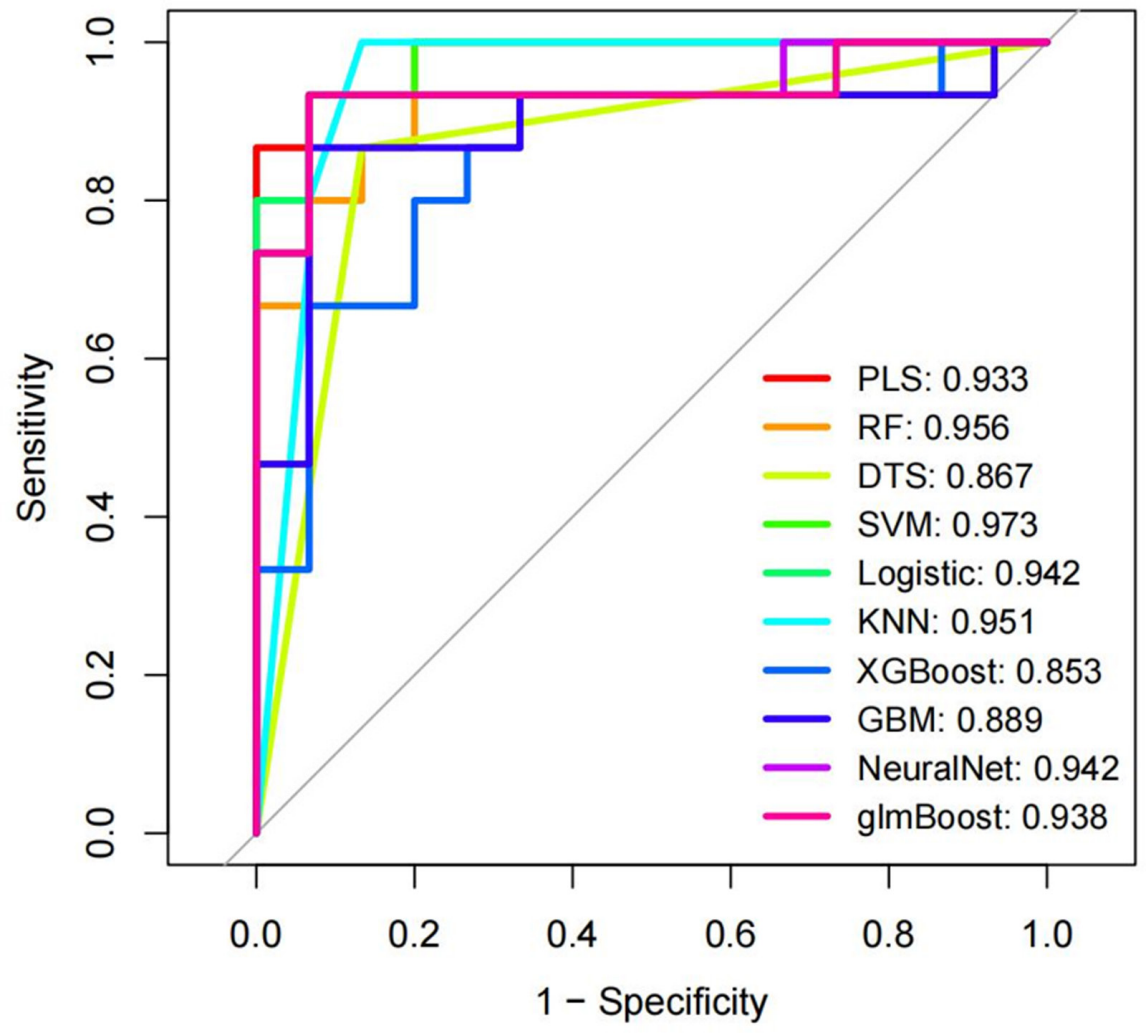
Evaluation of machine learning models. ROC curves of different machine learning models.

As illustrated in the figure, the curves of models such as RF, SVM, Logistic, *K*-nearest neighbor, and NeuralNet are positioned closer to the upper left corner, exhibiting higher AUC values. This indicates that these models achieve a superior balance between sensitivity and specificity, enabling more accurate classification of samples. Conversely, the curves of models such as DTS, XGBoost, and GBM are positioned lower, with lower AUC values, reflecting relatively weaker classification performance (Fig. 7).

### 3.6. SHAP interpretability analysis

**Figure 8.**
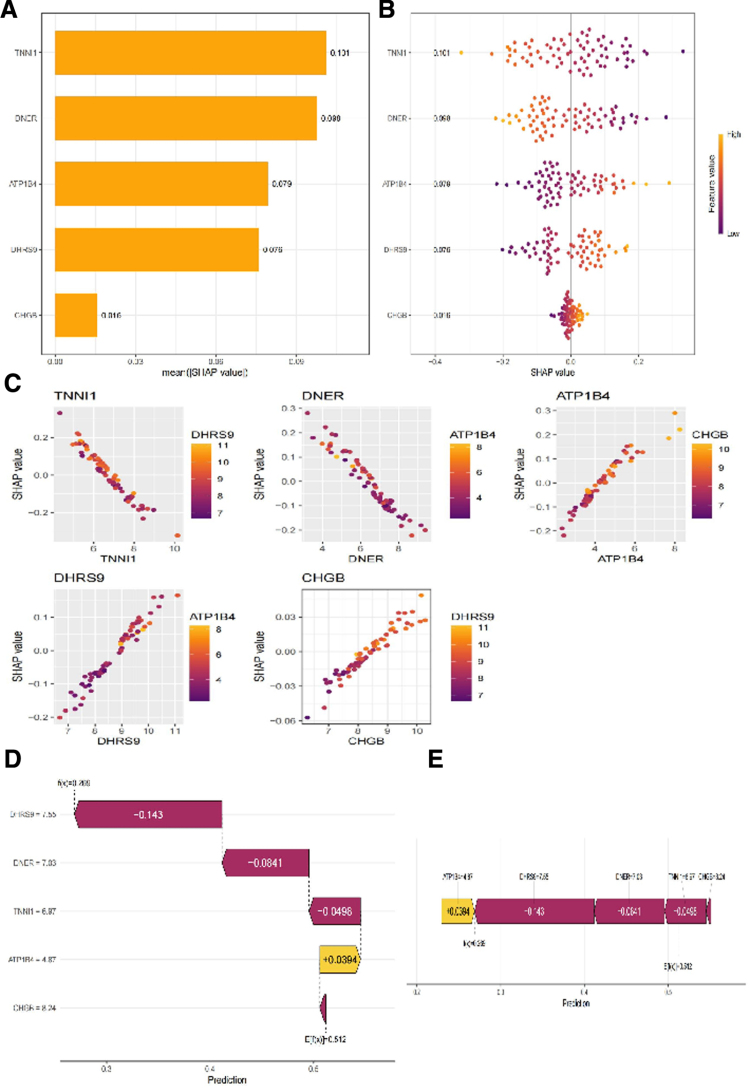
SHAP interpretability analysis. (A) SHAP feature importance bar plot; (B) SHAP summary plot; (C) SHAP dependence plots; (D) SHAP waterfall plot; (E) SHAP force plot. SHAP = shapley additive explanations.

SHAP analysis was employed to elucidate the decision-making process of machine learning models and quantify the contribution of each feature (gene) to the model’s prediction outcomes. The feature importance bar chart indicated that TNNI1 exhibited the largest average absolute SHAP value, playing a pivotal role in the model’s decision-making process. This was followed by DNER, ATP1B4, and DHRS9, while CHGB demonstrated the least influence. In the SHAP value scatter plot, high expression levels of TNNI1 and DNER positively influenced the model’s predictions, whereas the impact of ATP1B4, DHRS9, and CHGB varied depending on their respective expression values. The dependence plot further revealed that the positive contribution of high TNNI1 and DNER expression diminished with increasing expression levels, and ATP1B4, DHRS9, and CHGB exhibited complex nonlinear relationships with their expression values. The SHAP value summary plot illustrated the combined effects of multiple genes, such as the positive SHAP value of ATP1B4 enhancing the prediction score, while the negative SHAP values of DHRS9, DNER, and TNNI1 reduced it (Fig. 8A–E).

### 3.7. GSEA and GSVA analysis

**Figure 9.**
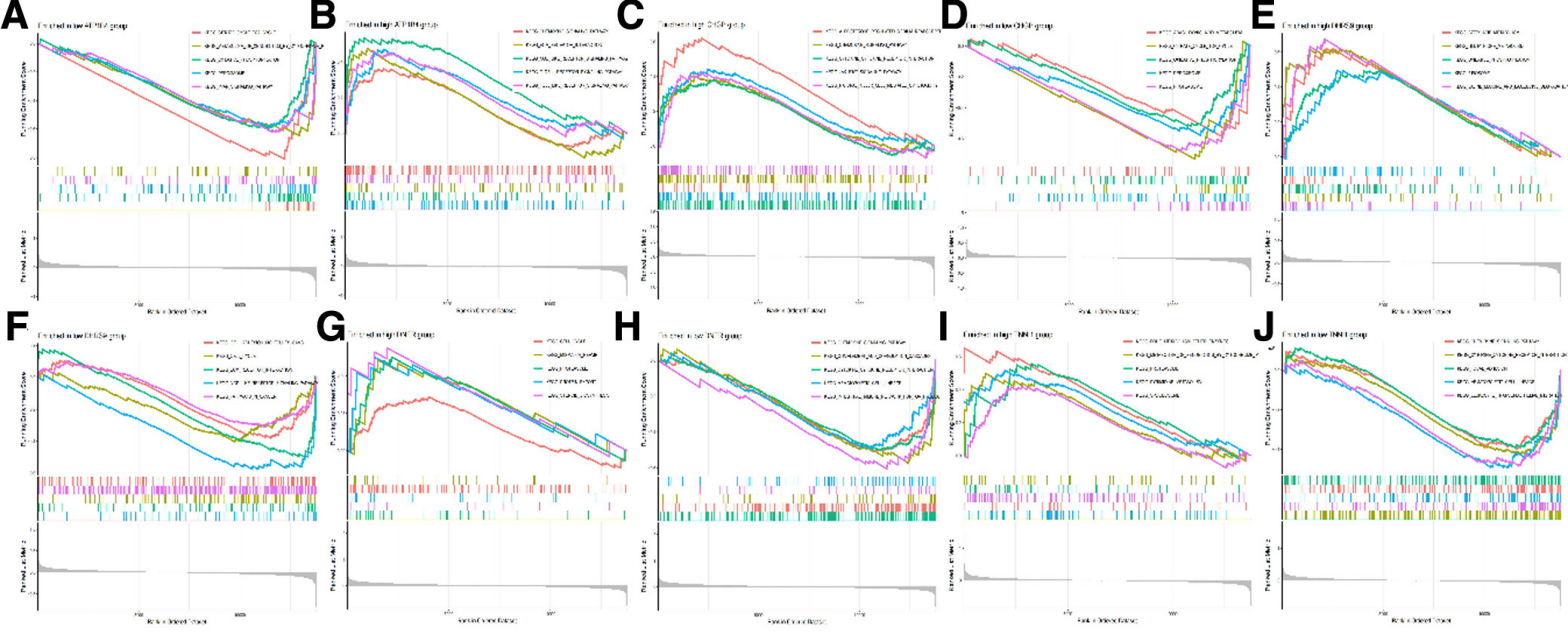
GSEA analysis of key feature genes. (A) GSEA plot for the ATP1B4 low-expression group; (B) GSEA plot for the ATP1B4 high-expression group; (C) GSEA plot for the CHGB high-expression group; (D) GSEA plot for the CHGB low-expression group; (E) GSEA plot for the DHRS9 high-expression group; (F) GSEAplot for the DHRS9 low-expression group; (G) GSEA plot for the DNER high-expression group; (H) GSEA plot for the DNER low-expression group; (I) GSEA plot for the TNNI1 high-expression group; (J) GSEA plot for the TNNI1 low-expression group. GSEA = gene set enrichment analysis.

This set of GSEA plots, based on gene expression levels, elucidates the enrichment patterns of KEGG pathways in different genes across high and low-expression groups. For ATP1B4, high expression is associated with the enrichment of chemokine signaling pathways involved in immune response and intercellular interaction, suggesting its role in immune regulation and cell adhesion. Conversely, low expression of ATP1B4 correlates with the enrichment of metabolic pathways, such as the citric acid cycle, highlighting its involvement in energy and material metabolism. High expression of CHGB is linked to pathways such as aldosterone-regulated sodium reabsorption, which are implicated in electrolyte balance and immune modulation; in contrast, low expression of CHGB is enriched in metabolic pathways like arachidonic acid metabolism, influencing metabolic regulation. DHRS9 exhibits involvement in fatty acid metabolism and other material metabolic processes when highly expressed, whereas low expression is associated with pathways such as cell adhesion molecules, which are related to cell proliferation and adhesion. DNER’s high expression is involved in the cell cycle and contributes to cell proliferation regulation and protein degradation; however, low expression is associated with immune response and coagulation processes. Finally, TNNI1 demonstrates involvement in material metabolism and protein degradation during high expression, while low expression is linked to immune regulation and cell adhesion (Fig. 9A–J).

**Figure 10.**
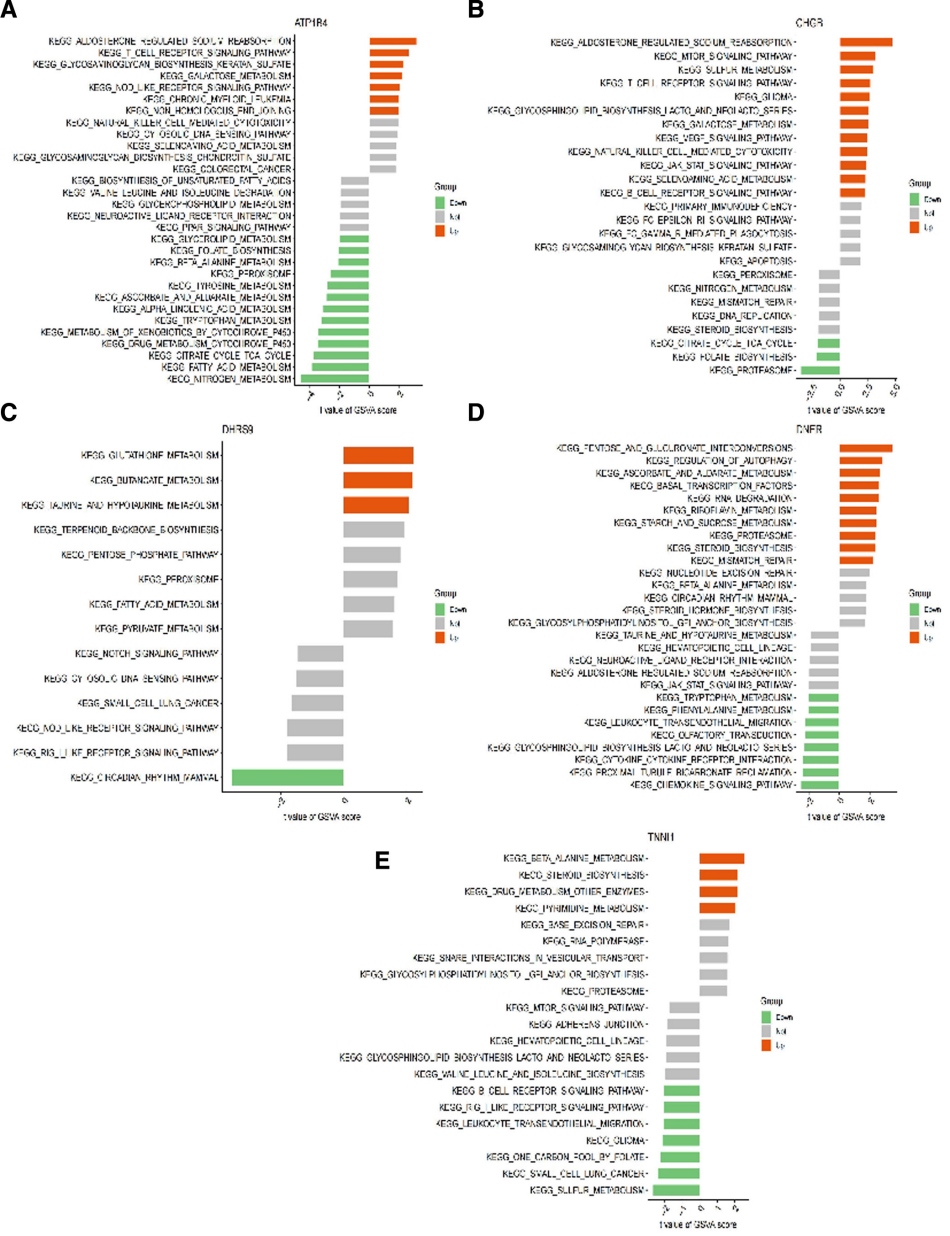
GSVA analysis of key feature genes. (A) GSVA score plot for ATP1B4; (B) GSVA score plot for CHGB; (C) GSVA score plot for DHRS9; (D) GSVA score plot for DNER; (E) GSVA score plot for TNNI1. GSVA = gene set variation analysis.

GSVA was performed on key genes, including ATP1B4 and CHGB, to elucidate their enriched KEGG pathways and GSVA score *t*-values. When ATP1B4 exhibited high expression levels, pathways associated with immune regulation and intercellular interaction were significantly upregulated, whereas those related to substance metabolism were markedly downregulated. Conversely, low expression of ATP1B4 resulted in the opposite pathway changes, underscoring its regulatory role in immunity and metabolism. High expression of CHGB was linked to pathways involved in electrolyte balance and cell growth regulation; however, low expression of CHGB led to inverse pathway alterations, reflecting its function in electrolyte balance and metabolic regulation. High expression of DHRS9 promoted pathways associated with substance metabolism, while low expression of DHRS9 induced opposing effects, suggesting its influence on substance metabolism and circadian rhythm regulation. High expression of DNER upregulated pathways related to substance metabolism and autophagy while downregulating immune-related pathways; conversely, low expression of DNER reversed these trends, emphasizing its importance in autophagy and immune regulation. High expression of TNNI1 upregulated pathways associated with substance metabolism, whereas low expression of TNNI1 resulted in inverse pathway changes, indicating its potential role in the regulation of substance metabolism (Fig. 10A–E).

### 3.8. Analysis of immune cell infiltration

**Figure 11.**
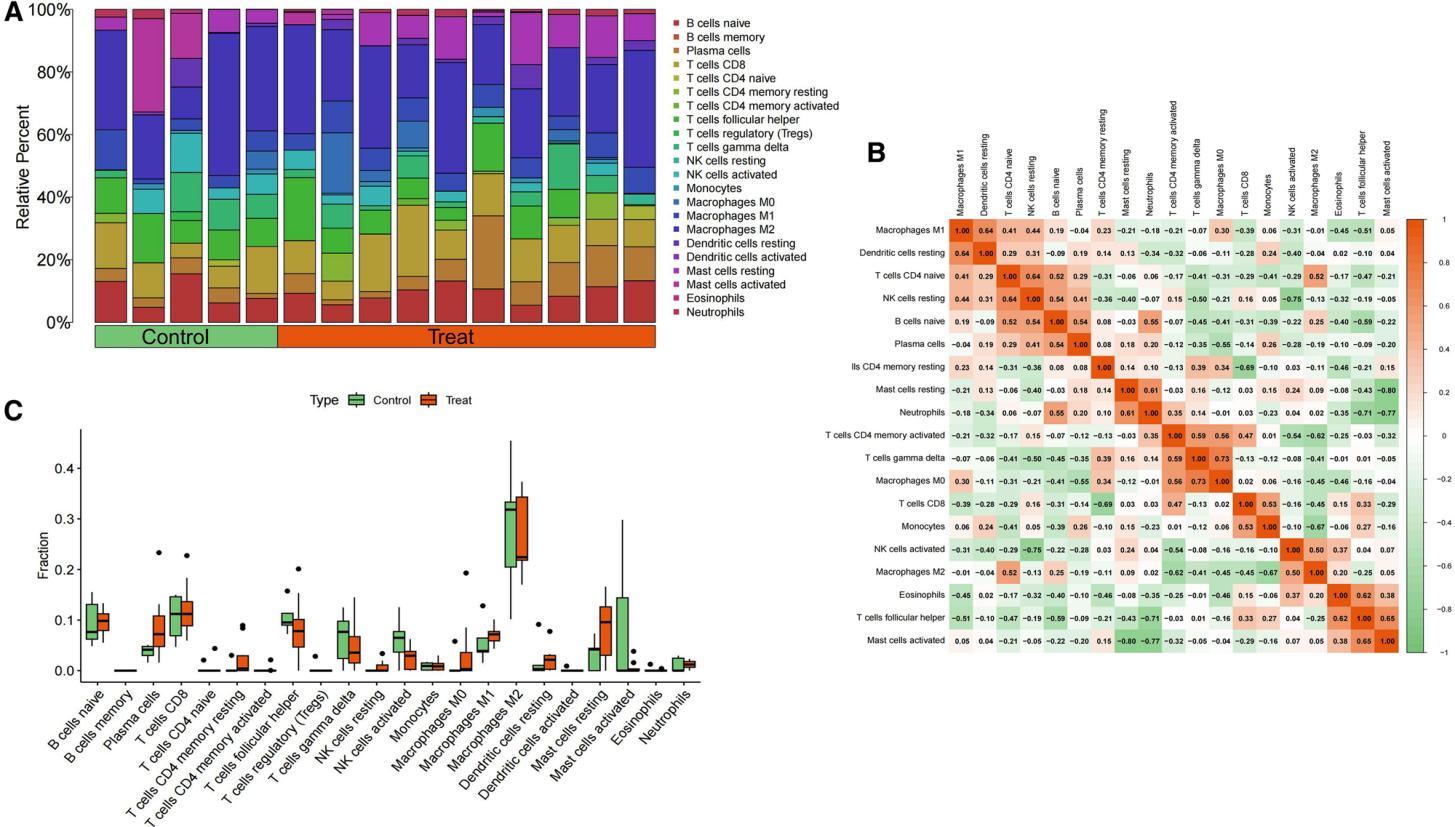
Analysis of immune cell infiltration. (A) Stacked bar plot of immune cell infiltration; (B) correlation heatmap of immune cells; (C) box plots of immune cell infiltration between control and treatment groups.

In this study, we employed stacked bar charts, correlation heatmaps, and box plots to comprehensively analyze the infiltration patterns of immune cells in both the control and experimental groups. The stacked bar charts systematically categorized various immune cell subtypes, including B cells, T-cells, NK cells, and their respective subpopulations. Upon comparing the data between the 2 groups, it was evident that the proportions of certain T cell and macrophage subgroups differed significantly, strongly indicating that the treatment in the experimental group influenced the composition of immune cells. The correlation heatmap visually represented the correlation coefficients between different immune cell types using a color gradient. Notably, macrophage M1 exhibited positive correlations with resting dendritic cells and naive CD4 T cells, while displaying negative correlations with eosinophils, thereby illustrating the intricate interaction network among immune cells. The box plots further highlighted the distribution of each immune cell subtype. A significant difference in the distribution of macrophage M0 was observed between the 2 groups, and the median and dispersion of activated mast cells also varied notably, providing robust evidence that the treatment in the experimental group had a substantial impact on the proportions of immune cells (Fig. 11A–C).

### 3.9. Correlation analysis of feature genes and immune infiltration

**Figure 12.**
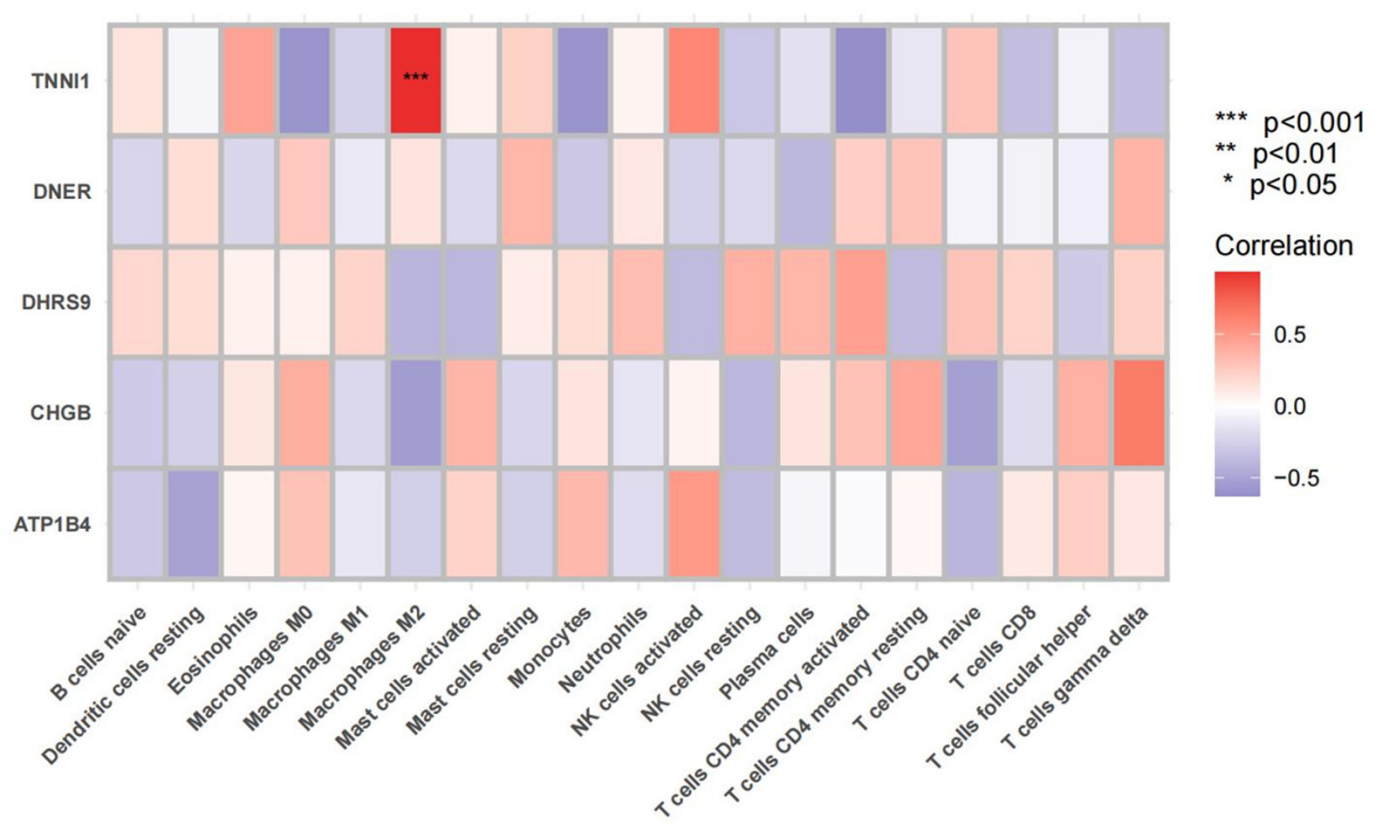
Correlation analysis between feature genes and immune cell infiltration. Correlation heatmap between key feature genes and immune cells.

This heatmap examines the correlation between feature genes, such as TNNI1, and immune cell infiltration. The color scheme provides relevant information: red indicates a positive correlation, with darker shades suggesting that the proportion or activity of immune cells is more likely to increase as gene expression rises; blue represents a negative correlation, with darker shades indicating that immune cell-related metrics are more likely to decrease as gene expression increases; white signifies no significant linear correlation. TNNI1 exhibits a significantly positive correlation with certain immune cells (*P* < .001), highlighting its critical role in influencing immune cell function or proportion. Additionally, DNER, DHRS9, CHGB, and ATP1B4 demonstrate varying degrees of correlation with immune cells. Although some correlations do not reach extremely high significance levels, they still suggest potential associations (Fig. 12).

## 4. Discussion

### 4.1. Biological significance of the unique gene expression profile in AF

Through the integration of multiple datasets, this study identified 6 DEGs, including 4 upregulated and 2 downregulated genes, thereby uncovering the distinct gene expression profile of AF patients. GO enrichment analysis revealed that these DEGs were significantly enriched in neuro-related biological processes, such as neuronal migration and glial cell differentiation, which aligns with the previously reported phenomenon of atrial neural remodeling in AF patients.^[17]^ Neural remodeling can disrupt normal electrical signal conduction, contributing to the onset and maintenance of AF. Abnormal expression of genes associated with neuronal migration may impair the coordination of atrial myocardial electrical activity, thereby increasing the risk of arrhythmias.^[18]^ Studies have demonstrated that nerve growth factor and its receptor TrkA are highly expressed in AF atrial tissue, driving excessive proliferation of sympathetic nerve fibers and inducing abnormal electrical activity.

KEGG pathway analysis indicated that the muscle cytoskeleton pathway was the most significantly enriched among DEGs, highlighting the critical role of myocardial cytoskeleton remodeling in AF. Previous studies have shown that abnormal expression of myocardial cytoskeleton proteins, such as α-actin and myosin heavy chain, during AF can lead to impaired myocardial contractile function and heterogeneous electrical conduction.^[19]^ Additionally, abnormal phosphorylation modification of the actin-binding protein α-actinin-2 can disrupt sarcomere structure, resulting in reduced atrial contractility.Furthermore, the aldosterone-regulated sodium reabsorption pathway was found to be enriched, consistent with the mechanisms underlying atrial fibrosis and ion homeostasis imbalance in AF patients. Aldosterone promotes myocardial sodium overload by activating the epithelial sodium channel, leading to calcium homeostasis disorders and delayed after depolarization. This has been confirmed in rabbit AF models, where aldosterone antagonists significantly improved atrial electrical remodeling.^[20]^

In this study, only 6 DEGs meeting the prespecified thresholds were identified under the multi-cohort integration framework – a finding consistent with our analytical principle of prioritizing cross-cohort reproducibility. Compared with single-cohort differential analysis, integrative analysis of cross-platform and cross-source samples – following rigorous batch effect correction and quantile normalization – tends to retain differential signals exhibiting both directional consistency and robust effect sizes across independent cohorts. In contrast, genes showing cohort-specific significance, inconsistent fold changes, or opposing expression directions are more likely to be attenuated in meta-analytic settings due to statistical averaging and stringent multiple-testing control. Concurrently, we applied conservative filtering criteria (FDR < 0.05 and |log_2_FC|>1), along with low-expression gene removal and post-integration normalization – measures that collectively minimize false discovery in public data integration, albeit at the cost of reduced sensitivity for marginally significant transcripts. Batch effects were corrected using ComBat (sva package), and PCA was employed solely to assess batch correction efficacy by visualizing pre-versus post-correction sample clustering – not for batch correction or model training.

Given the well-documented heterogeneity across AF cohorts – including variations in clinical phenotypes, anatomical sampling sites, and tissue cellular composition – many transcriptional changes are inherently subtype-or context-dependent. When such heterogeneity structures differ across cohorts, integrative analysis does not aim to maximize DEG count but rather converges on a high-confidence, biologically coherent core set – the “reproducible differential axis” – that reflects fundamental, shared pathobiological mechanisms. To confirm that the limited DEG yield reflects biological stringency rather than methodological artifact, we conducted threshold sensitivity analyses: relaxing FDR or log_2_FC cutoffs markedly increased DEG yield, whereas the 6 retained genes demonstrated consistent up- or down-regulation directionality across ≥ 4 of the 5 integrated cohorts – supporting their status as robust, cross-cohort anchors.

Although the number of DEGs is small, they exhibit strong biological coherence and functional interpretability. First, they collectively enable accurate discrimination between AF and non-AF samples at the global transcriptomic level, confirming their capacity to encode stable, phenotype-associated signals. Second, GO and pathway enrichment analyses converge on biologically plausible, AF-relevant modules – including neural regulation and autonomic remodeling, myocardial structural and cytoskeletal reorganization, ion channel homeostasis, and hormonal signaling – all of which align mechanistically with established paradigms of electrical and structural remodeling in AF. Third, machine learning models built upon these features achieve high classification performance (AUC ≥ 0.85, with the best-performing model reaching AUC > 0.92), and SHAP-based interpretability analysis confirms their consistent, non-redundant contributions across individual samples. Critically, pathway-level enrichment patterns show concordant associations with immune cell infiltration profiles derived from CIBERSORTx, suggesting that this compact DEG set may represent pivotal regulatory nodes at the intersection of immune–metabolic activation and structural remodeling.

Notably, the GO term enrichment for “neural-related processes” is interpreted cautiously: it likely reflects transcriptional reprogramming associated with autonomic nervous system remodeling – such as altered expression of neurotransmitter receptors, neuropeptides, or neural – cardiac coupling factors – rather than the physical presence of neuronal or glial cells in atrial tissue. These signatures may arise from cardiomyocytes, fibroblasts, or infiltrating immune cells responding to neurohumoral stress, and thus represent functional neuroregulatory states within the cardiac microenvironment. Their precise cellular origin and causal directionality remain to be resolved using spatially resolved or single-cell transcriptomic approaches.

### 4.2. Functional validation of key feature genes and their association with disease

The 6 key feature genes (DHRS9, TNNI1) identified via machine learning algorithms exhibited significant expression differences in AF patients. DHRS9, as an upregulated gene, is associated with the activation of the fatty acid metabolism pathway and may play a role in myocardial energy metabolic remodeling during AF.^[21]^ Research has demonstrated that the metabolic shift from fatty acid oxidation to glucose utilization in myocardial cells of AF patients is abnormal, and DHRS9 may influence myocardial cell function by modulating lipid metabolism.^[22]^ In mouse models, overexpression of DHRS9 enhances fatty acid uptake in myocardial cells, leading to mitochondrial dysfunction and increased oxidative stress, which aligns with the pathological hallmark of myocardial energy depletion observed in AF. TNNI1, as a downregulated gene, encodes cardiac troponin I (cTnI), a critical regulator of myocardial contraction. Reduced expression of TNNI1 may result in diminished myocardial contractility, consistent with the clinical observation of decreased atrial contractile function in AF patients.^[23]^ Clinical studies have revealed that the phosphorylation level of cTnI in atrial tissue of AF patients is significantly reduced, accompanied by a decline in myofilament calcium sensitivity. Furthermore, SHAP analysis indicated that TNNI1 contributed most significantly to model prediction, with its expression positively correlated with immune cell infiltration, suggesting that TNNI1 may be involved in the AF pathological process through immune–myocardial interactions. Animal experiments have shown that cTnI released from myocardial injury can activate dendritic cells, induce Th17 cell differentiation, and exacerbate atrial inflammation.^[24]^

### 4.3. Potential mechanisms of immune–metabolic interactions in AF

GSEA and GSVA analyses revealed that characteristic genes were implicated in the progression of AF through the regulation of immune modulation, metabolic pathways, and autophagy. When ATP1B4 was highly expressed, it exhibited enrichment in the chemokine signaling pathway, suggesting its potential role in recruiting immune cells to induce atrial inflammation. Conversely, when ATP1B4 expression was low, it was associated with energy metabolism regulation. As the β4 subunit of the sodium–potassium pump, high ATP1B4 expression enhances the secretion of T-cell chemokine (C-X-C Motif Chemokine Ligand 10), thereby promoting CD4 + T-cell infiltration – a phenomenon observed in patients with rheumatic heart disease complicated by AF.^[25]^ This aligns with the “immune–metabolic imbalance” hypothesis in AF pathogenesis: inflammatory cytokines (e.g., interleukin-6) inhibit mitochondrial pyruvate dehydrogenase activity in cardiomyocytes, while metabolic disturbances (e.g., insufficient glucose utilization) activate the NOD-like receptor pyrin domain-containing protein 3 inflammasome, further exacerbating immune cell activation.^[26]^ Immune cell infiltration analysis demonstrated that the proportion of M0 macrophage subgroups was significantly altered in the AF group, with a complex association between these changes and the expression of characteristic genes. Macrophages contribute to atrial fibrosis and electrical remodeling by secreting pro-inflammatory cytokines such as tumor necrosis factor-α and interleukin-6.^[27]^ Genes like DHRS9 may influence AF progression by regulating macrophage polarization (M1/M2 type conversion). In AF mouse models, inhibiting DHRS9 reduces M1-type macrophage infiltration and alleviates atrial fibrosis.^[28]^ Additionally, the positive correlation between TNNI1 and T-cell subsets implies that TNNI1 released from damaged cardiomyocytes may serve as an antigen to trigger adaptive immune responses, thereby exacerbating atrial pathological alterations.^[29]^ Clinical studies have confirmed that autoantibody levels against cTnI are significantly elevated in the peripheral blood of AF patients and correlate with disease severity.

### 4.4. Clinical translational value of machine learning models

The RF, SVM, and other machine learning models developed in this study demonstrated high classification efficacy (AUC ≥ 0.85), with TNNI1, DHRS9, and other characteristic genes serving as the core predictive factors. These findings provide potential biomarker combinations for noninvasive diagnosis of AF. The expression ratio of DHRS9 to TNNI1 may act as a molecular indicator for assessing the risk of AF occurrence. A multicenter study involving 1200 patients revealed that the blood-based DHRS9/TNNI1 ratio was positively correlated with AF recurrence (HR = 1.87, 95% confidence interval: 1.24–2.83, *P* < .01).^[30]^ Furthermore, SHAP analysis elucidated the dynamic influence of gene expression on model prediction. When TNNI1 expression was elevated, its positive contribution to AF prediction diminished, suggesting the presence of an expression threshold effect. This insight offers a novel perspective for monitoring AF progression. Model predictions indicated that when TNNI1 expression fell below the threshold (log2[transcripts per kilobase of exon model per million mapped reads] = 3.2), the risk of AF significantly increased, aligning with clinical observations that patients with severe myocardial injury are more susceptible to AF.^[31]^

### 4.5. Research limitations and future directions

This study relies on the analysis of public datasets, lacking prospective clinical validation and in-depth mechanistic exploration through animal experiments. Future investigations should employ single-cell sequencing to elucidate the cell-specific expression patterns of characteristic genes within atrial tissue, determine whether ATP1B4 is predominantly expressed in cardiomyocytes or infiltrating immune cells, and validate its functional role using gene knockout models. In AF mouse models, myocardial-specific knockout of ATP1B4 has been shown to significantly reduce the AF induction rate.^[32]^ Furthermore, the interaction network between immune cells and cardiomyocytes requires further clarification through co-culture experiments and proteomics analysis. Recent studies utilizing spatial transcriptomics technology have demonstrated that the spatial proximity of macrophages and cardiomyocytes in AF atrial tissue is positively correlated with the extent of fibrosis,^[33]^ underscoring the critical importance of intercellular communication in AF pathogenesis.

## 5. Conclusion

Through the integration and comprehensive analysis of multiple datasets, this study has elucidated the gene expression characteristics and molecular mechanisms underlying AF. A total of 6 DEGs were identified (four upregulated: DHRS9, CHGB, ATP1B4, RELN; 2 downregulated: TNNI1, DNER), exhibiting distinct expression patterns in AF patients. Functional enrichment analysis revealed that these genes were significantly enriched in neural-related biological processes, muscle cytoskeleton pathways, and aldosterone-regulated sodium reabsorption pathways. These findings suggest that neural remodeling, abnormal myocardial cytoskeleton structure, and ion homeostasis imbalance are critical factors contributing to the onset of AF. Furthermore, machine learning algorithms identified 5 key feature genes whose expression differences were closely associated with myocardial energy metabolism remodeling and contractile dysfunction in AF patients. These genes formed a complex regulatory network by modulating immune regulation, metabolic pathways, and autophagy. Additionally, the constructed machine learning model demonstrated that the expression ratio of DHRS9 to TNNI1 could serve as a potential biomarker for noninvasive diagnosis of AF, offering a novel tool for clinical application.

## Author contributions

**Conceptualization:** Wen Bai.

**Data curation:** Mierzhati Maimaiti, Aizizha paerhati, Wen Bai.

**Formal analysis:** Mierzhati Maimaiti.

**Funding acquisition:** Xianglin Du, Wen Bai.

**Investigation:** Mierzhati Maimaiti, Aizizha paerhati, Shenhong Liu, Xianglin Du, Wen Bai.

**Methodology:** Mierzhati Maimaiti, Aizizha paerhati, Shenhong Liu, Wen Bai.

**Project administration:** Xianglin Du.

**Resources:** Aizizha paerhati, Shenhong Liu, Wen Bai.

**Software:** Mierzhati Maimaiti, Aizizha paerhati, Xianglin Du.

**Supervision:** Mierzhati Maimaiti, Aizizha paerhati, Shenhong Liu, Wen Bai.

**Validation:** Mierzhati Maimaiti, Shenhong Liu, Xianglin Du, Wen Bai.

**Visualization:** Shenhong Liu, Xianglin Du.

**Writing – original draft:** Mierzhati Maimaiti.

**Writing – review & editing:** Wen Bai.


