# Supplementary material for: Research on identification of key genes and immune–metabolic mechanisms in atrial fibrillation through integrated multi-cohort transcriptomic analysis and machine learning
Source: Medicine (Baltimore). 2026 Jun 26;105(26):e49463. doi: 10.1097/MD.0000000000049463 (PMC13313671; doi:10.1097/MD.0000000000049463)
Supplement: Supplementary file 1 [file medi-105-e49463-s001.doc]

**Table S1. Summary of included GEO datasets**

| **GEO accession** | **Platform (GPL)** | **Platform type** | **Tissue source** | **Group definition (AF subtype)** | **Sample size (AF / Control)** |
| --- | --- | --- | --- | --- | --- |
| GSE31821 | Affymetrix Human Genome U133 Plus 2.0 Array (GPL570) | mRNA expression microarray | Atrial/auricle tissue biopsy | AF vs sinus rhythm (SR)/controls | 4 vs 2 |
| GSE79768 | Affymetrix Human Genome U133 Plus 2.0 Array (GPL570) | mRNA expression microarray | Paired left and right atrial specimens | Persistent AF vs SR | 13 vs 13 patients; paired LA/RA, 26 arrays total |
| GSE108660 | Agilent-062918 OE Human lncRNA Microarray V4.0 (GPL19612) | lncRNA expression microarray (including mRNA probes) | Right atrial tissue/right atrial appendage | Permanent AF (rheumatic valvular heart disease background) vs SR | 5 vs 5 |
| GSE115574 | Affymetrix Human Genome U133 Plus 2.0 Array (GPL570) | mRNA expression microarray | Left and right atrial tissues | Permanent AF vs SR (severe MR background) | 15 vs 15 patients; 59 arrays in GEO |
| GSE143924 | Affymetrix Human Gene 2.0 ST Array (GPL25483) | mRNA expression microarray | Epicardial adipose tissue (EAT) biopsy | Postoperative AF (POAF; postoperative new-onset/short-term event) vs non-POAF/controls | 15 vs 15 |
